# Prevalence of the anterosuperior capsulolabral anatomical variations and their association with pathologies of the glenoid labrum: a systematic review and meta-analysis

**DOI:** 10.1007/s00402-023-04932-9

**Published:** 2023-06-23

**Authors:** Michal Benes, David Kachlik, Lubomir Kopp, Vojtech Kunc

**Affiliations:** 1 Department of Anatomy, Second Faculty of Medicine, Charles University, Prague, Czech Republic; 2 Clinic of Trauma Surgery, Masaryk Hospital, Usti nad Labem, Czech Republic

**Keywords:** Sublabral recess, Sublabral foramen, Buford complex, SLAP lesion, Glenoid labrum

## Abstract

**Purpose:**

Differentiating the anatomical variations of the anterosuperior portion of the glenoid labrum from pathologies is important to avoid unnecessary iatrogenic complications resulting from inaccurate diagnosis. Additionally, the presence of several variations was reported to be conductive to lesions involving the glenoid labrum. Thus, the aim of this study was to state the prevalence rates of the sublabral recess, sublabral foramen, and the Buford complex, and to verify their association with labral lesions.

**Methods:**

Systematic search of electronic databases was conducted to gain potentially eligible literature. Suitable studies were selected in a two-round screening, and relevant data were subsequently extracted. Calculation of the pooled prevalence estimates, including sub-analyses on cohort size, study type, and geographical variance, was conducted. Pooled analysis of risk ratios (RR) was used to assess the conductive nature of the discussed variants to superior labrum anterior to posterior (SLAP) lesions.

**Results:**

The screening resulted in selection of 20 studies investigating the morphological features of the glenoid labrum, consisting of 7601 upper limbs. On the bases of random-effects meta-analysis the sublabral recess, sublabral foramen and Buford complex occur with a pooled prevalence of 57.2% (95% CI 30.0–84.4%), 13.5% (95% CI 8.2–18.9%), and 3.0% (95% CI 1.5–4.5), respectively. Moreover, individuals with Buford complex have RR 2.4 (95% CI 1.3–4.7) of developing SLAP lesions, especially type II (95.5%; 95% CI 86.1–100%), whereas such risk for sublabral recess and sublabral foramen was not statistically significant.

**Conclusion:**

Morphological variants of the glenoid labrum posing diagnostic confusion are frequently observed. Gradually, the Buford complex may be a predisposing factor for sustaining a SLAP lesion.

## Introduction

The glenoid labrum is considered as one of the most important contributors to the static stabilization of the glenohumeral joint [21]. Morphologically, it consists of a peripheral fibrous layer and a transitional fibrocartilaginous layer (zone). Both of these layers provide insertion points for different anatomical structures around the shoulder girdle. Mostly, the peripheral layer serves as an anchor for the tendon of the long head of the biceps brachii muscle as well as for the glenohumeral ligaments and joint capsule. Contrary, the transitional layer provides fine attachment of the aforementioned peripheral layer to the deeper and central parts of the glenoid cavity [13, 20].

Lesions involving the glenoid labrum are common pathological conditions. Especially, injuries of the superior portion of the labrum are nowadays observed with rising incidence in throwing athletes and individuals with a history of fall on an outstretched hand with adducted and flexed arm [18, 22]. These lesions were closely studied by Snyder et al. [22], who called them the superior labrum anterior and posterior (SLAP) lesions since they begin posteriorly and extend anteriorly to the middle part of the glenoid with or without the involvement of the origin of the tendon of the long head of the biceps brachii muscle. Moreover, Snyder et al. [22] classified the SLAP lesions based on their extent as fraying of the superior portion of the labrum with degenerative appearance (type I), tearing with detachment of the origin of the tendon of the long head of the biceps brachii muscle (type II), a bucket-handle displacement with an intact origin of the long head of the biceps brachii muscle (type III), and a bucket-handle fragment extending to the tendon of the long head of the biceps brachii muscle (type IV). Although several extensions have been proposed, the original classification by Snyder et al. [22] is still generally accepted [15].

**Fig. 1:**
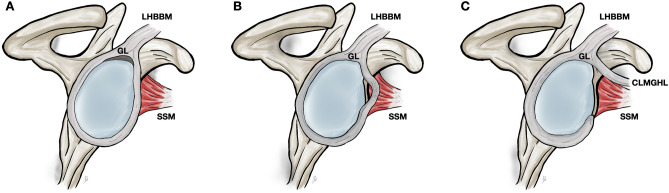
Schematic drawings of the sublabral recess (**A**), the sublabral foramen (**B**), and the Buford complex (**C**). CLMGHL cord-like middle glenohumeral ligament, GL glenoid labrum, LHBBM long head of the biceps brachii muscle, SSM subscapularis muscle

The diagnostic evaluation of the anterosuperior portion of the glenoid labrum remains challenging and requires profound knowledge of the shoulder anatomy [25]. Therefore, the SLAP lesions may be mistaken for normal anatomical variations of the glenoid labrum and vice versa [10, 21]. With heterogeneous prevalence rates the sublabral recess, sublabral foramen, and the so-called Buford complex have been reported to cause diagnostic confusions. The sublabral recess (*recessus sublabralis*) represents a loose attachment of the superior portion of the labrum on the glenoid cartilage (Fig. 1A) [24, 27]. The sublabral foramen (*foramen sublabrale*), also termed the sublabral hole, is defined as a window-like structure bordered by the normal anterosuperior labrum and the anterior cartilaginous margin of the glenoid cavity (Fig. 1B) [2, 13, 25]. The Buford complex, first described by Williams et al. [25] is identified upon three criteria: (1) a cord-like middle glenohumeral ligament (MGHL) continuous with anterosuperior portion of the labrum is present; (2) the combined MGHL and anterosuperior portion of the labrum is attached to the superior labrum at the base of the tendon of the long head of the biceps brachii muscle; and (3) no additional anterosuperior labral tissue is present, giving the appearance of a large void below or posteromedial to the cord-like MGHL (Fig. 1C). In addition, it has been reported that the presence of the labral anatomical variations is correlated with higher occurrence of SLAP lesions compared to control groups with regular anatomy [13, 16].

In view of the clinical importance of the anatomical variations observed in the anterosuperior capsulolabral complex, this study aims to create pooled prevalence data on the sublabral recess, sublabral foramen and Buford complex with the use of random-effects meta-analysis, and to verify their conductive attributes to lesions of the glenoid labrum.

## Materials and methods

The study protocol was prospectively registered on PROSPERO under the identification number CRD42022356234. Preferred Reporting Items for Systematic Reviews and Meta-Analyses (PRISMA) 2020 guidelines were consciously followed throughout the study process [17].

### Search strategy

A structured search of major scientific databases, including Web of Science, PubMed, ScienceDirect, Scopus, SciELO, JSTOR and Google Scholar, was performed from inception through March 2022. The MeSH terms “Buford complex”, “sublabral foramen”, “sublabral hole”, “foramen sublabrale”, “sublabral recess”, “recessus sublabralis”, “glenoid labrum”, “variability”, and “anatomy” were selected and used in combinations to compile all relevant studies. A representative example of combinations used for Web of Science is attached in Table 1. No restrictions on the language or document type were applied. The reference lists of published articles were also checked for any missed studies.

**Table 1:** Example of search term combinations used for Web of Science

Search number	Combination
1	Buford complex (topic)
2	glenoid labrum (topic) and variability (topic) or anatomy (topic)
3	sublabral foramen (topic) or sublabral recess (topic) or sublabral hole (topic)
4	foramen sublabrale (topic) or recessus sublabralis (topic)

### Data extraction and eligibility criteria

The data were extracted in a two-round screening provided by two reviewing authors (M.B. and V.K.). Citation manager Mendeley v. 1.19.4 (Elsevier, UK) was used for data curation in both rounds of screening. Initially, titles with abstracts were reviewed and all apparently irrelevant hits were excluded. Then, in the second round, the remaining articles were assessed for eligibility by inspecting the full-texts. Only original studies on humans were considered suitable for the meta-analysis. The inclusion criteria met cadaveric studies with natural anatomy, as well as individuals from clinically oriented studies with possible pathological conditions. Review articles, case reports, conference abstracts, studies on animals, and studies containing incomplete or misleading data unresolved by email correspondence were excluded. In case of studies with overlapping data, larger cohort size and relevance of information was the decisive for inclusion. All studies that were not written in English were translated by professionals fluently speaking the particular language. Information on demographics, sample size, year of publication, incidence of the discussed anatomical variations, and eventually the associations between labral morphological variants and labral lesions were extracted. Attempt to contact the original corresponding authors of selected studies was made when uncertainties were recognized during the data extraction.

### Quality assessment

Each paper was independently evaluated by two authors (M.B. and V.K.) for risk of bias assessment. Due to the anatomical nature of this meta-analysis the Anatomical Quality Assessment (AQUA) Tool was used as a guide for quality appraisal [8]. This checklist consists of five domains focusing on objectives, study design, methodology, descriptive anatomy, and reporting of results, with each domain having a set of signaling questions. The risk of bias is judged as low if all questions are answered with “Yes”. Contrary, questions answered with “No” might indicate the potential source of bias. All unclear findings were resolved by a reached consensus between all authors. Funnel plots were also used to determine the possibility of bias.

### Statistical analysis

The software R v. 4.1.1 (R Project for Statistical Computing, Austria) with RStudio 1.4.1717 (RStudio, USA) was used to analyze all obtained input data. Calculation of pooled prevalence estimates was achieved using the random-effects model. The frequency of the distinct variants and the overall sample size of each included study was used as input data for determining the pooled prevalence estimates. To measure the effect size 95% confidence intervals (95% CI) were calculated. To evaluate the heterogeneity between the included studies Cochran’s Q test and Higgins *I*^2^ statistics were used. The *P* value of < 0.10 was a priori set as “significant heterogeneity”. Values for *I*^2^ were categorized as “might not be important” between 0 and 40%, “may indicate moderate heterogeneity” between 30 and 60%, “may represent substantial heterogeneity” between 50 and 90%, and “may represent considerable heterogeneity” between 70 and 100% [9]. Confidence intervals were also used to state statistical significance in terms of their absent overlap when comparing two outcomes. To further investigate the sources of heterogeneity and probe for sources of bias, sub-analyses minding the cohort size, study type, and geographical origin were conducted. Potential of small sample size bias was assessed by visual inspection of funnel plots. Additionally, meta-analysis of binary outcomes was used for inspection of associated pathologies with the morphological variations. Risk ratio (relative risk, RR) was calculated for each of the relevant studies and the individual RRs were consequently pooled for cumulative analysis. Confidence intervals of the calculated RRs were used to determine the statistical significance.

## Results

### Identification of studies

Systematic search of databases yielded a total of 1471 hits, and after checking for duplicates 846 hits remained. In the first round of screening, comprising title and abstract checking, 801 hits were excluded. Full-text screening resulted in 15 studies meeting the inclusion criteria. Additional five studies were added from reference checking. Therefore, a total of 20 studies were finally deemed eligible for inclusion in the meta-analysis. See flowchart displayed in Fig. 1 for detailed overview of the study selection process.

### Characteristics of studies

**Fig. 2:**
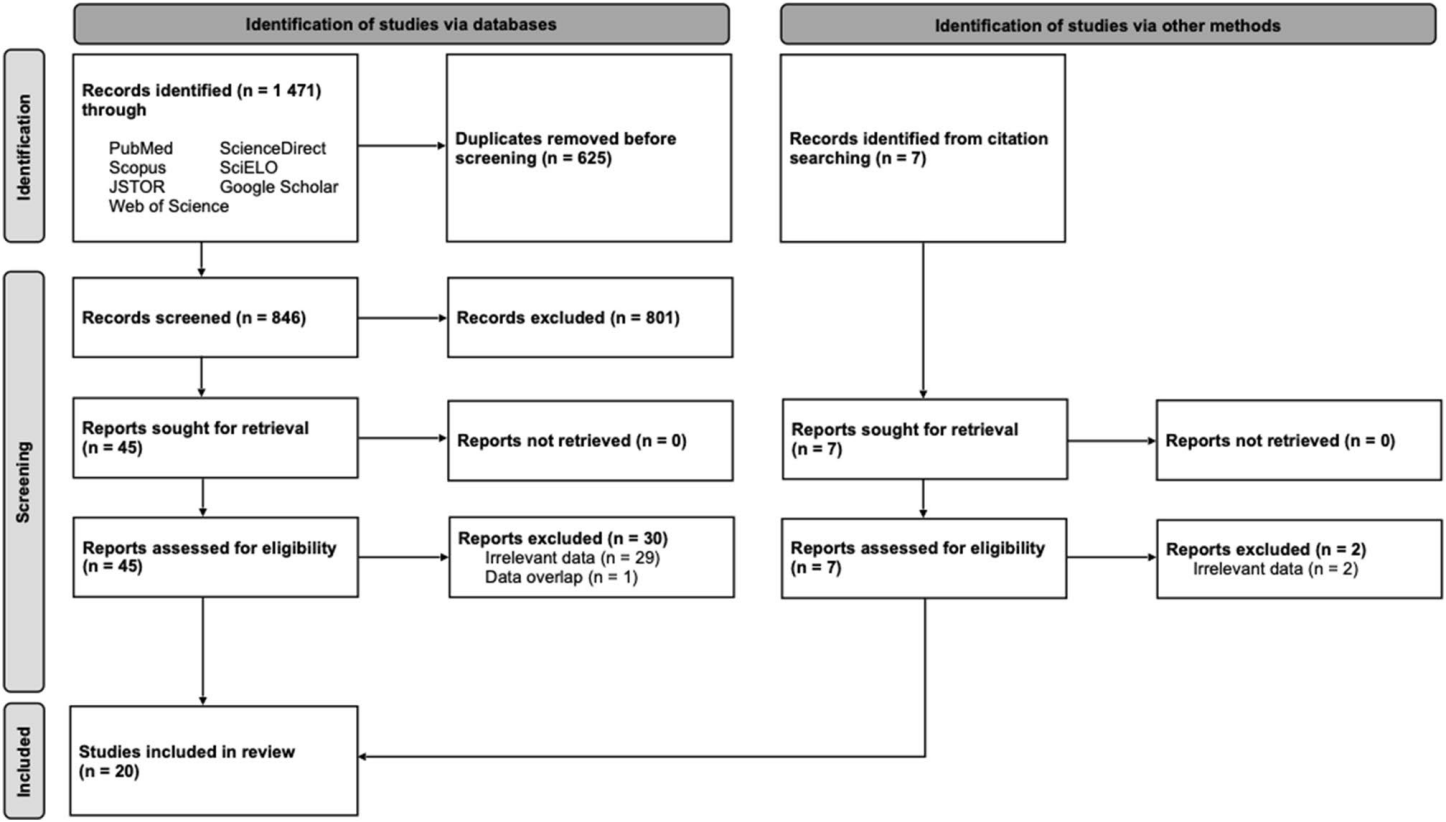
PRISMA 2020 flow diagram

Overall, 20 studies were used for meta-analysis with a total number of upper limbs of 7601 and average cohort of 380.1 cases (range 15–3, 129). Geographically, ten studies originated from North America, five from Asia, four from Europe, and one from Australia. From the total of 20 studies, ten were arthroscopic, six were cadaveric, and four were radiological studies. Among the included studies, twelve of them were evaluated as having high probability of bias according to the AQUA Tool. Study characteristics with risk of bias assessment are summarized in Table 2. Based on the electronic communication with the corresponding authors, the study by Ozer et al. [16] includes patients from the study by Kanatli et al. [13]. Therefore, the study by Kanatli et al. [13] was omitted from the meta-analysis on the Buford complex, but yet this study is included in the analyses regarding the sublabral recess and sublabral foramen since these data do not duplicate with the latter study. Also, study by Ilahi et al. [12] overlaps with a newer study published by the same author in 2008 [11]. The former study was therefore excluded from the analysis (Fig. 2).

**Table 2:** Study characteristics

Study	Country	Type	Cohort size	Risk of bias
Bachler et al., 2020 [1]	France	Arthroscopic	300	Low
Bain et al., 2012 [2]	Australia	Cadaveric	19	High
Bents and Skeete, 2005 [3]	USA	Arthroscopic	235	Low
Connell et al., 1999 [4]	USA	Radiological	140	Low
Handelberg et al., 1998 [6]	Belgium	Arthroscopic	530	High
Harzmann et al., 2003 [7]	Germany	Cadaveric	20	High
Ide et al., 2004 [10]	Japan	Cadaveric	84	High
Ilahi et al., 2008 [11]	USA	Arthroscopic	334	High
Kanatli et al., 2010 [13]	Turkey	Arthroscopic	691	Low
Kaptan et al., 2022 [14]	Turkey	Arthroscopic	809	Low
Ozer et al., 2021 [16]	Turkey	Arthroscopic	3129	Low
Pappas et al., 2013 [18]	USA	Cadaveric	102	High
Park et al., 2000 [19]	Korea	Radiological	108	High
Rao et al., 2003 [20]	USA	Arthroscopic	546	High
Shortt et al., 2009 [21]	USA	Radiological	88	Low
Thompson et al., 2015 [23]	USA	Radiological	104	Low
Waldt et al., 2006 [24]	Germany	Cadaveric	43	High
Williams et al., 1994 [25]	USA	Arthroscopic	200	High
Wilson et al., 2013 [26]	USA	Arthroscopic	104	High
Yeh et al., 1998 [27]	USA	Cadaveric	15	High

### Sublabral recess

**Fig. 3:**
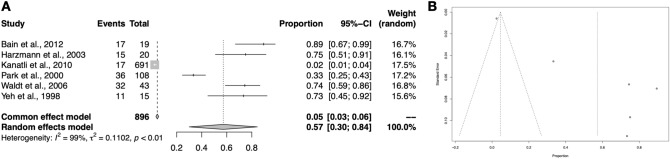
Forest plot (**A**) and funnel plot (**B**) regarding the meta-analysis on the sublabral recess

A total of six studies (896 upper limbs) reported relevant data on the sublabral recess. The analysis revealed that the pooled prevalence estimate is 57.2% (95% CI 30.0–84.4%) (Fig. 3). Both sensitivity analysis and visual inspection of funnel plot indicate that this outcome is biased by the small sample. In general, sub-analyses including studies with larger cohort sizes showed that the actual prevalence might be significantly lower – in extreme situations as low as 2.5% (95% CI 1.3–3.6%) found in an arthroscopic study, which was conducted on a considerable sample. Detailed results are attached in Table 3.

**Table 3:** Detailed overview of the performed sub-analyses

Analysis	Sublabral recess			Sublabral foramen			Buford complex		
	Prevalence (95% CI)	*I*^2^	*P*	Prevalence (95% CI)	*I*^2^	*P*	Prevalence (95% CI)	*I*^2^	*P*
Overall	57.2% (30.0–84.4%)	98.7%	< 0.01	13.5% (8.2–18.9%)	93.7%	< 0.01	3.0% (1.5–4.5%)	88.0%	< 0.01
Sensitivity < 100	79.2% (70.3–88.1%)	3.1%	0.38	14.5% (4.5–24.5%)	86.0%	< 0.01	1.8% (0–3.5%)	0%	0.48
Sensitivity ≥ 100	17.6% (0–47.8%)	97.8%	< 0.01	13.2% (6.6–19.9%)	94.7%	< 0.01	3.7% (1.4–6.1%)	91.7%	< 0.01
Athroscopic	2.5% (1.3–3.6%)	NA	NA	10.9% (6.2–15.6%)	93.5%	< 0.01	2.6% (1.2–4.0%)	92.3%	< 0.01
Cadaveric	79.2% (70.3–88.1%)	3.1%	0.38	16.7% (6.2–27.3%)	90.0%	< 0.01	1.9% (0–3.8%)	2.6%	0.40
Radiological	33.3% (24.4–42.2%)	NA	NA	15.0% (0–32.0%)	95.9%	< 0.01	8.5% (0–19.9%)	92.3%	< 0.01
Asia	17.6% (0–47.8%)	97.8%	< 0.01	4.8% (0–10.0%)	94.6%	< 0.01	2.9% (1.1–4.7%)	76.6	< 0.01
Australia	89.5% (7.0–75.7%)	NA	NA	26.3% (6.5–46.1%)	NA	NA	NA	NA	NA
Europe	74.6% (63.9–85.4%)	0	0.96	13.6% (5.1–22.0%)	0	0.41	0.5% (0–1.2%)	0	0.46
North America	73.3% (51.0–95.7%)	NA	NA	15.7% (8.2–23.2%)	95.0%	< 0.01	5.1% (1.2–9.0%)	84.9%	< 0.01

Not applicable (NA) is reported in instances of missing data for the particular sub-analysis caused by only a single study belonging to the sub-group or absence of a relevant study in the particular sub-group

### Sublabral foramen

**Fig. 4:**
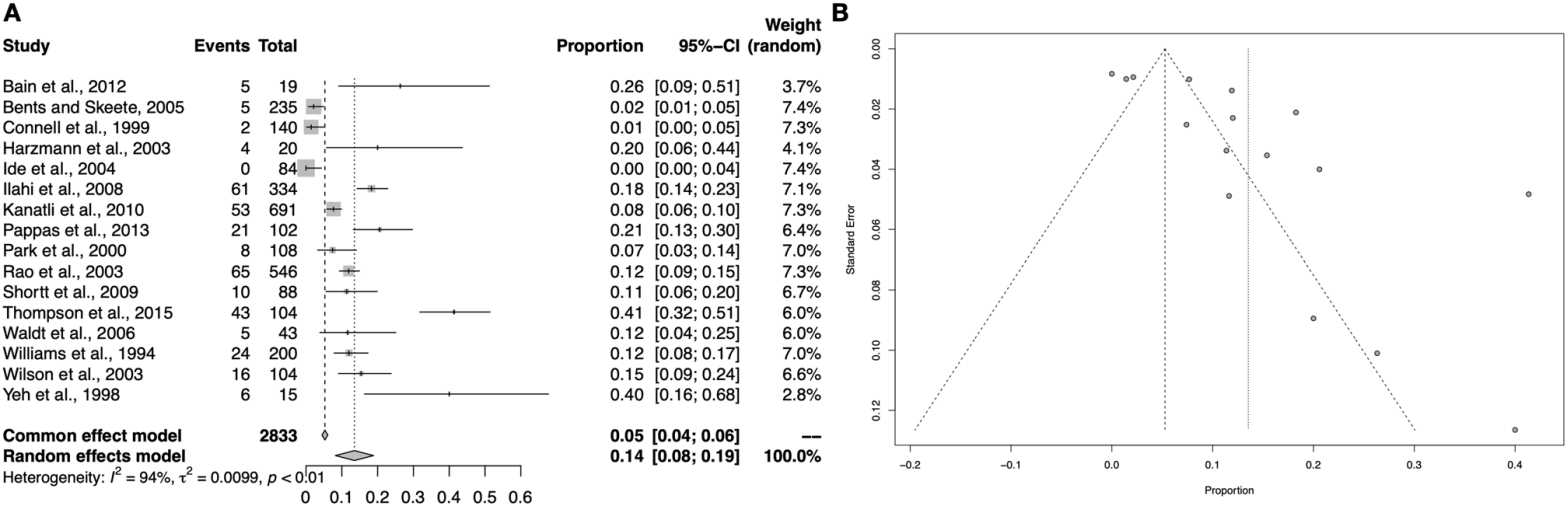
Forest plot (**A**) and funnel plot (**B**) regarding the meta-analysis on the sublabral foramen

The sublabral foramen was described in 16 studies (2833 upper limbs), and occurs with a pooled prevalence of 13.5% (95% CI 8.2–18.9%). Pooling of the closer morphological findings reported in three studies showed that in 67.2% (95% CI 55.2–79.1%) of cases the foramen existed together with a cord-like MGHL [11, 20, 25]. Throughout the performed sub-analyses the prevalence rates remained nearly identical to the overall analysis with the exception of geographical disparity in Asia and Australia (Table 3). Nevertheless, these sub-analyses include limited sample (Fig. 4).

### Buford complex

**Fig. 5:**
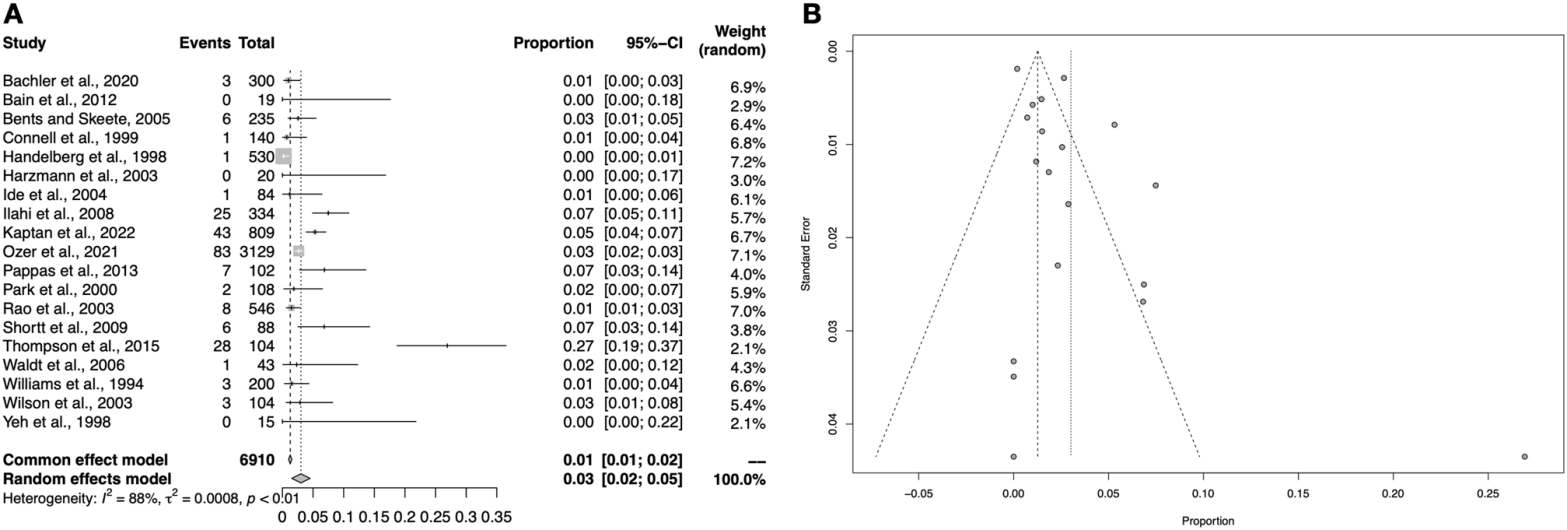
Forest plot (**A**) and funnel plot (**B**) regarding the meta-analysis on the Buford complex

In total, 19 studies (6910 upper limbs) contained relevant data on the Buford complex. The overall pooled prevalence was calculated to be 3.0% (95% CI 1.5–4.5) (Fig. 5). Higher prevalence rates were found among radiological studies, followed by arthroscopic and cadaveric studies, respectively. Simultaneously, the Buford complex was found more prevalently, although insignificantly, in North America, followed by Asia and Europe, respectively. See Table 3 for detailed results of the performed analyses.

### Association with labral pathologies

**Fig. 6:**
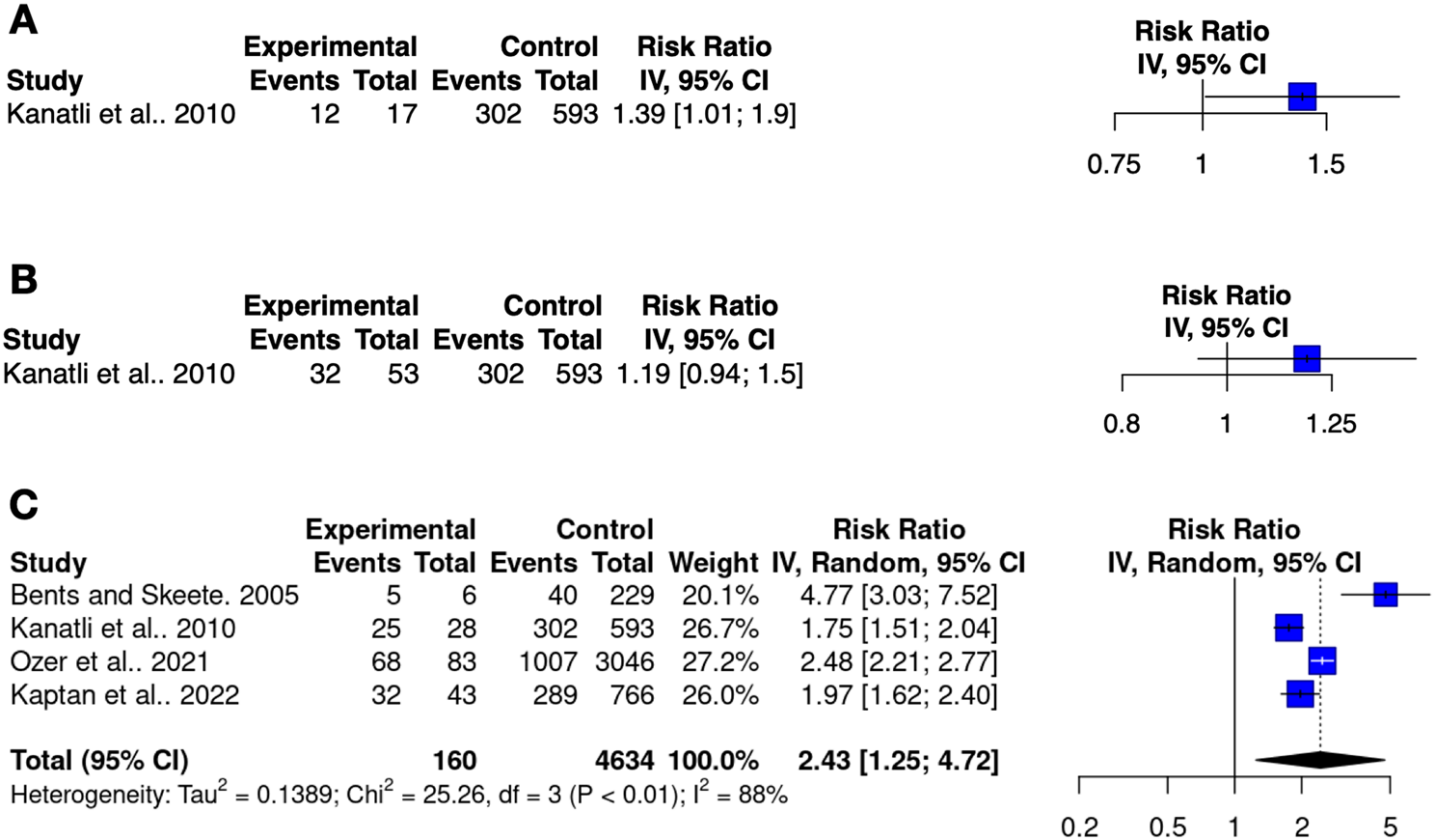
Forest plot of risk ratios (RR) regarding the meta-analysis on the association of sublabral recess (**A**), sublabral foramen (**B**), and Buford complex (**C**) with SLAP lesions

The association of the anterosuperior capsulolabral variations with SLAP lesions was discussed in four arthroscopic studies (4864 upper limbs) [3, 13, 14, 16]. However, the relationship with sublabral recess and sublabral foramen was observed only in one study [13]. All four studies provided sufficient data for exploring the association of SLAP lesions and the presence of the Buford complex. Conclusively, the sublabral recess and sublabral foramen do not significantly increase the risk of SLAP lesions with RR of 1.4 (95% CI 1.0–1.9) and 1.2 (95% CI 0.9–1.5), respectively (Fig. 6A, B). Conversely, individuals with Buford complex have RR of 2.4 (95% CI 1.3–4.7) of developing a SLAP lesion (Fig. 6C). Most commonly the Buford complex occurs with type II lesions (95.5%; 95% CI 86.1–100%), followed by type I (3.2%; 95% CI 0–10.8%) and type III (< 0.1%; 0–1.9%), respectively.

## Discussion

Morphological variations of the anterosuperior capsulolabral complex must be differentiated from lesions of the glenoid labrum and adjacent glenohumeral ligaments. Their relatively common appearance detected in the presented study should be consciously taken in mind during diagnostic shoulder arthroscopies to obtain the correct diagnosis and avoid preventable complications. The presence of the Buford complex may predispose to SLAP lesions. Such outcomes are of particular interest when evaluating the shoulder joint for pathology as the presence of the Buford complex may lead the surgeon towards proper exploration of the anterosuperior portion of the glenoid labrum, where, in particular, type II SLAP lesions occur.

Pooled analysis indicated that the overall prevalence rates for the sublabral recess, sublabral foramen, and Buford complex are 57.2%, 13.5%, and 3.0%, respectively. Nevertheless, further research is required in the field of the sublabral recess since the number of large cohort studies is limited. As the results of the sub-analyses are quite heterogeneous, this probably signifies reporting bias. On the other hand, the equivocal outcomes may also support high variability between research methods or geographical variance. For the reason of insufficient data, the association of sublabral recess and SLAP lesions is only theoretical.

During a shoulder arthroscopy the sublabral recess can resemble a small irregularity of the superior or anterosuperior labral insertion onto the glenoid and therefore can be misdiagnosed as a small labral tear [2, 7]. Compared to SLAP lesions, the sublabral recess is shallow and usually does not extend dorsally over the attachment of the tendon of the long head of the biceps brachii muscle [7, 24]. Nevertheless, extension to the posterior segment of the labrum may occur, which poses diagnostic confusions [24]. Morphologically, the sublabral recess features synovial lining [2]. Performing an excess anterior stabilization of the sublabral recess may limit the patient slightly in his shoulder range of motion.

The sublabral foramen is frequently observed together with the cord-like MGHL (67.2%). Knowledge of this co-incidence is definitively useful when intraoperatively testing the attachment of the glenoid labrum. Nevertheless, the presence of the sublabral foramen was not deemed indicative of pathology as the function of the glenoid labrum is not impaired [24]. This statement is believed to be true in case of intact glenohumeral ligaments and muscles forming the rotator cuff. Only a single study investigating the association of the sublabral foramen and SLAP lesions was identified for our analysis, and the outcomes are insignificant of the conductive nature. The arthroscopic picture of a sublabral foramen can imitate a limited anterior or anterosuperior labral tear (soft Bankart lesion or SLAP lesion), sometimes even forming a communication with the subscapular bursa. Compared to the traumatic lesion, the sublabral foramen is of limited size (up to 1–2 cm), has smoother margins without synovial reaction and is not displaced from the glenoid. A misleading stabilization of this normal labral variant can lead to slight limitation of shoulder range of motion, especially in external rotation.

Conversely, the Buford complex was positively correlated with higher risk of SLAP lesions. The outcomes of our study show that the RR was 2.4 in individuals with Buford complex. Ozer et al. [16] concluded that the risk is raised due to a repetitive micro-trauma and secondary acute trauma explained by increased load bearing on the superior portion of the labrum proximal to the MGHL as the missing anterosuperior portion of the labrum associated with the Buford complex alters physiological distribution of forces applied on the circumferential glenoid labrum. Therefore, the superior labrum is prone to injury due to the applied stress. Compared to the previous variants, the Buford complex can be very convincing during the arthroscopic surgery, resembling severe anterosuperior labral tear (SLAP lesion). With the absence of normal glenolabral insertion between the 1 and 3 o’clock, thickening of the MGHL and direct superior labral insertion onto the subscapularis muscle tendon, it can mimic a major pathology in ventral shoulder chamber. An erroneous anterior stabilization of this fully physiological variant can lead to severe intraoperative damage to anterior joint capsule, incorrect stabilization of subscapularis muscle tendon to anterior part of the glenoid, and produces a severe limitation of shoulder range of motion, especially in abduction, external rotation and dorsal flexion. It belongs to the most debilitating iatrogenic surgical consequences in arthroscopic shoulder surgery.

As mentioned above, individuals with Buford complex have higher risk of developing a SLAP lesion. Therefore, a novel technique modifying the anatomy of the Buford complex in SLAP lesion repairs has been proposed [5]. Apart from the standard SLAP repair, the cord-like MGHL is transected, and the proximal remnant is tightly affixed to the supraglenoid tubercle, while the distal remnant is left intact within the joint. By this change of former morphology, the release of MGHL prevents unwanted pulling on the superior portion of the glenoid labrum and the attachment of the tendon of the long head of the biceps brachii muscle. Performing such a procedure should avoid recurrence of the SLAP lesion in patients with Buford complex, who naturally have higher risk of sustaining this type of injury. From the perspective of intermediate follow-up, this technique has shown satisfactory improvement in outcomes [5]. Significant improvements were reported in motion, pain relief, strength, and subjective satisfaction. Noteworthy, surgical interventions in symptom-free patients with Buford complex should not be performed due to a risk of development of shoulder stiffness [25].

The anatomical variations of our interest have also been discussed as contributing factors to conditions other than the SLAP lesions. Rao et al. [20] compared a group of patients with sublabral foramen occurring with and without the cord-like MGHL, and Buford complex with control group composed of patients with regular anatomy. They found statistically significant association with tears of the tendon of the subscapularis muscle as well as labral fraying. Possible explanation was the increased range of internal rotation in the group of patients with the variants, which predisposes to anterosuperior or coracoid impingement. On the other hand, a significantly lower prevalence of tears of the tendon of the supraspinatus and infraspinatus muscles was observed in the group of patients with abnormal anatomy. This is presumed to be caused by unknown biomechanical alterations that play a protective role. Unfortunately, this study could not be included in our analysis because the data regarding the distinct variants are unextractable for our purposes. However, these findings should encourage researchers to elaborate further on the clinical associations with the intra-articular variations.

Throughout all morphological analyses a high heterogeneity between the data persisted. This can be explained by several aspects. First, a high variance across continents may exist. Second, the methods used to assess the variants feature ranging sensitivity, and thus can be missed especially in the radiological evaluations. And lastly, the limited sample size used for the investigations may not be representative of general population. We believe this is the reason of substantial discrepancy between the sub-analyses performed on the prevalence of the sublabral recess in particular. However, these limiting factors were adjusted in the individual sub-analyses. The arthroscopic studies pose another limitation. It must be borne in mind that the patients underwent arthroscopy due to a symptomatology, and therefore were not randomly selected from the general population. Perhaps, this group may have different prevalence rates of the variants as shown in the sub-analyses minding the study types. To definitively confirm the conductive nature of the Buford complex to sustaining SLAP lesion, prospectively designed observational studies comprising individuals with Buford complex are needed.

## Conclusion

Morphological variations in the anterosuperior capsulolabral complex, including the sublabral recess, sublabral foramen and Buford complex, are frequently observed with estimated prevalence of 57.2%, 13.5%, and 3.0%, respectively. These variants must be carefully differentiated from pathological conditions. Moreover, the Buford complex may play a conductive role to SLAP lesions, especially type II. Knowledge of such association should be borne in mind while arthroscopically exploring the shoulder joint for impairments.

## Data Availability

Data are available on request to corresponding author.
